# A study to investigate the effectiveness of the application of virtual reality technology in dental education

**DOI:** 10.1186/s12909-022-03543-z

**Published:** 2022-06-15

**Authors:** Meysam Siyah Mansoory, Seyyed Mohsen Azizi, Fakhrosadat Mirhosseini, Danial Yousefi, Hedaiat Moradpoor

**Affiliations:** 1 Department of Biomedical Engineering, School of Medicine, Kermanshah University of Medical Sciences, Kermanshah, Iran; 2 Medical Education and Development Center, Arak University of Medical Sciences, Arak, Iran; 3 Trauma Research Center & Educational Development Center, Kashan University of Medical Sciences (KaUMS), Kashan, Iran; 4 Department, School of Allied Medical Sciences, Kashan University of Medical Sciences, Anesthesia, Kashan, Iran; 5 Department of Computer Engineering, Islamic Azad University of Central Tehran Branch, Tehran, Iran; 6 Department of Prosthodontics, School of Dentistry, Kermanshah University of Medical Sciences, Kermanshah, Iran

**Keywords:** Virtual reality, Dental education, Neutral zone, Teeth arrangement

## Abstract

**Background:**

Today, the use of virtual reality (VR) technology as an educational tool in dental education has expanded considerably. This study was aimed to evaluate the effectiveness of using VR technology in teaching neutral zone and teeth arrangement.

**Methods:**

This randomized trial was conducted at Kermanshah University of Medical Sciences, Iran in 2019. The study sample consisted of 50 six-year dental students who were randomly divided into experimental (*n* = 25) and control (*n* = 25) groups. Students’ performance in both groups was assessed using tests. A questionnaire was used to assess the usability of VR technology and students’ satisfaction with it.

**Results:**

All faculty members confirmed the usability of VR technology in dental education. The majority of students (76%) were highly satisfied with the use of this technology in their learning process. The mean score of students was significantly higher in the experimental group (16.92 ± 1.12) than in the control group (16.14 ± 1.18).

**Conclusion:**

In general, it can be argued that VR technology is useful and effective in the teaching–learning process. Therefore, its use in medical and dental schools can play an effective role in creating a dynamic, attractive, and successful learning environment.

## Introduction

Nowadays, the potential application of digital technologies in the teaching–learning process has drawn the attention of many scientific fields [1–4]. One of these new technologies is virtual reality (VR) [5, 6]. This technology has also been used in medical and dental education and is referred to as a teaching tool [5]. The VR technology represents the artificial simulation of a real-life environment using a computer. This technology can put the user in a position to feel they are in the real world [7].

Dentistry, as one of the most important fields of health sciences in the world, has a high potential for the use of digital technologies such as VR [8]. The use of new learning approaches and technologies such as VR has become a necessity for the departments of dentistry in universities around the world [8]. In this regard, the results of a systematic review showed that VR technology is an opportunity for a revolution in the field of dental education and can play an important role in the future of this field [9]. One of the necessities of using VR technology is that it is difficult for dental students to observe and study oral anatomy in the real world. In this regard, the results of a study showed that the use of VR technology can improve dental students’ understanding of anatomical interactions and can create an attractive learning environment for them [10].

The common techniques used for teaching neutral zone design and teeth arrangement at Iranian dental schools are the face-to-face and demonstration techniques, through which the basics of prosthodontics are conveyed to the students, and students can practice on their models.

This training protocol has been used for many years. However, it has several problems such as teacher’s dependency on the students, a possible loss of some important points, viewing the training process just from one angle, unrepeatability of the necessary sessions, and teaching several complex techniques in a single session. Furthermore, the students may undergo anxiety and stress during teaching, which may negatively affect their learning process.

To overcome these educational problems, VR technology and 3-dimensional (3D) computer models and simulators can be considered appropriate options for teaching the treatment used for edentulous patients. This study was principally aimed to assess the effect of using VR on teaching the procedures of neutral zone design and teeth arrangement in complete dentures, a topic that has not been investigated so far.

### Study aims

1. Assessing the usability of virtual reality technology in dental education from the perspective of faculty members
2. Assessing students' satisfaction with the use of virtual reality technology in dental education
3. Compare the effect of instruction based on virtual reality and instruction based on lecture on dental students’ learning outcome

## Materials and methods

This randomized trial was designed and conducted at the School of Dentistry, Kermanshah University of Medical Sciences in 2019. The statistical population included all dental students (*n* = 50) at Kermanshah University of Medical Sciences. The sample size was obtained based on Siyah Mansoory et al. study [5] and using Stata software (version 12) at 95% confidence level and 80% power of 21 people in each group. Therefore, in order to increase the statistical power, the sample size was increased to 50 people. All students were enrolled in the study by census method and were randomly divided into experimental (*n* = 25) and control (*n* = 25) groups. We used Permuted block randomization method for random allocation of samples [11]. The inclusion criteria were passing at least two semesters and willingness to participate in the study. One of the most important features in interventional studies is blinding. Blinding increases the validity of the results and reduces the bias [12]. According to the purpose of the present study, it was not possible to blind faculty members and participants.

### Procedures

Data collection was performed in two phases. The first phase was the design of the virtual reality environment, and the second phase was the implementation and data collection.

### Phase one

**Fig. 1:**
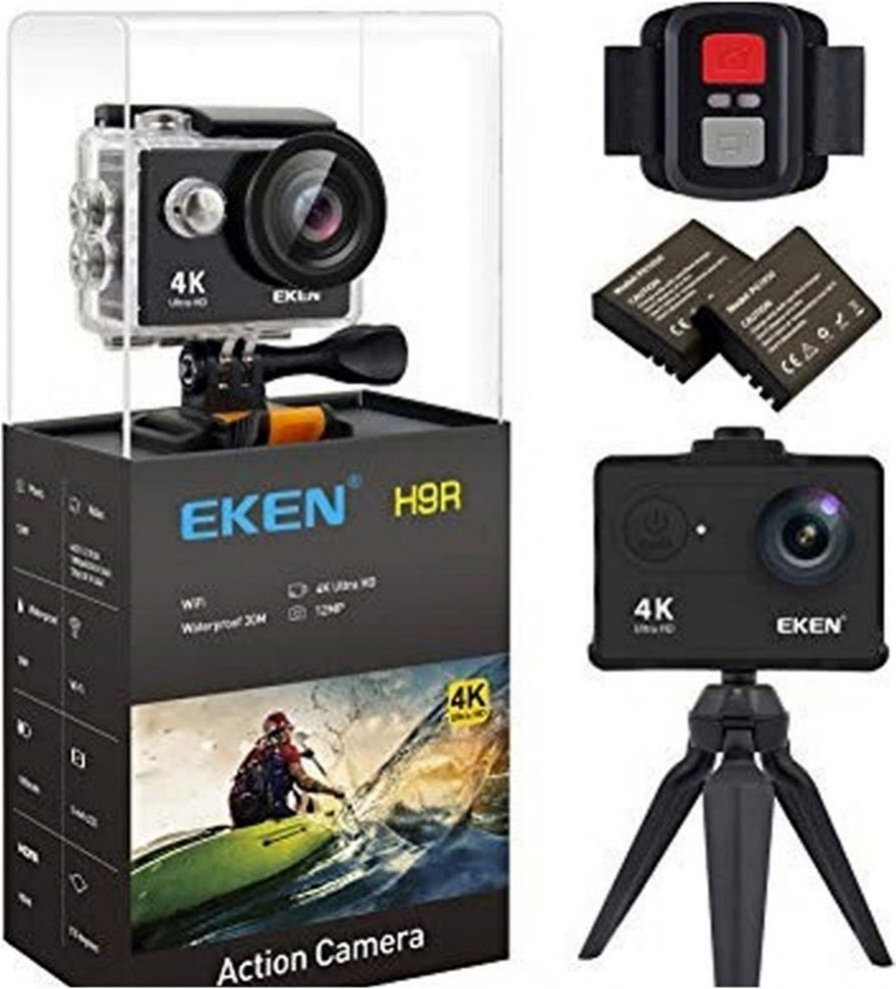
Imaging technology used in the study

**Fig. 2:**
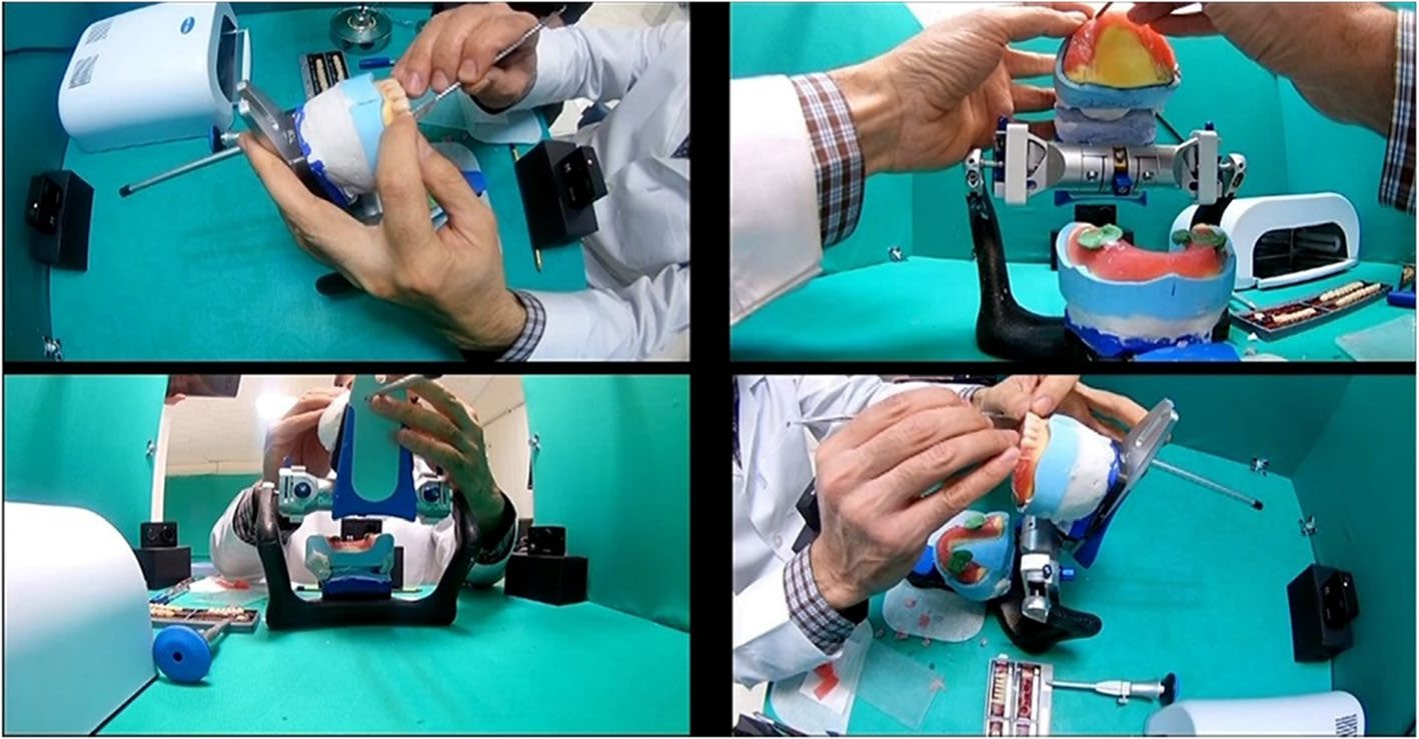
Imaging of teeth arrangement

After the preliminary studies and group discussions with concerned specialists, the multi-view video capturing method was used to implement the VR system, in which five EKEN 4KUHD 60 frames-per-second cameras instantly filmed the given procedure from different views during the prosthodontist’s demonstration (Figs. 1 and 2). Filming was also initiated simultaneously by the radio remote control. Afterward, the videos were filmed in a small portable studio, manufactured based on the green screen technology (Chroma Key).

These films were then fed into the Unity game engine, following which a 3D environment was applied to the training procedure.

**Fig. 3:**
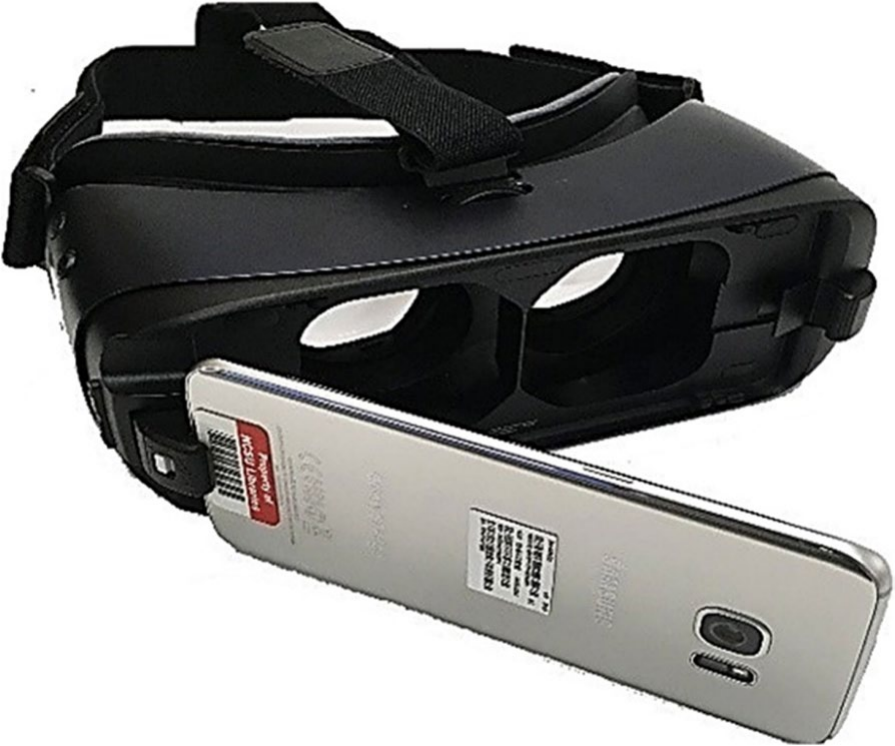
The virtual reality headset used in the study

Notably, the user could watch the training procedure from different angles by moving in the virtual space. Further, the programming environment was created by the Unity game engine, which was then operated with the android system 5 and above. The headset used in this study was of the Gear VR type, manufactured by Samsung Company in cooperation with Oculus Company (owned by Facebook). Moreover, this headset was programmed by the Oculus VR framework. In addition to the cost factor, the biggest advantages of using Gear VR are its wireless property and the ability to install a cellphone on the headset, which enables the students to view the educational procedures by placing their cellphones in the headset (Fig. 3).

**Fig. 4:**
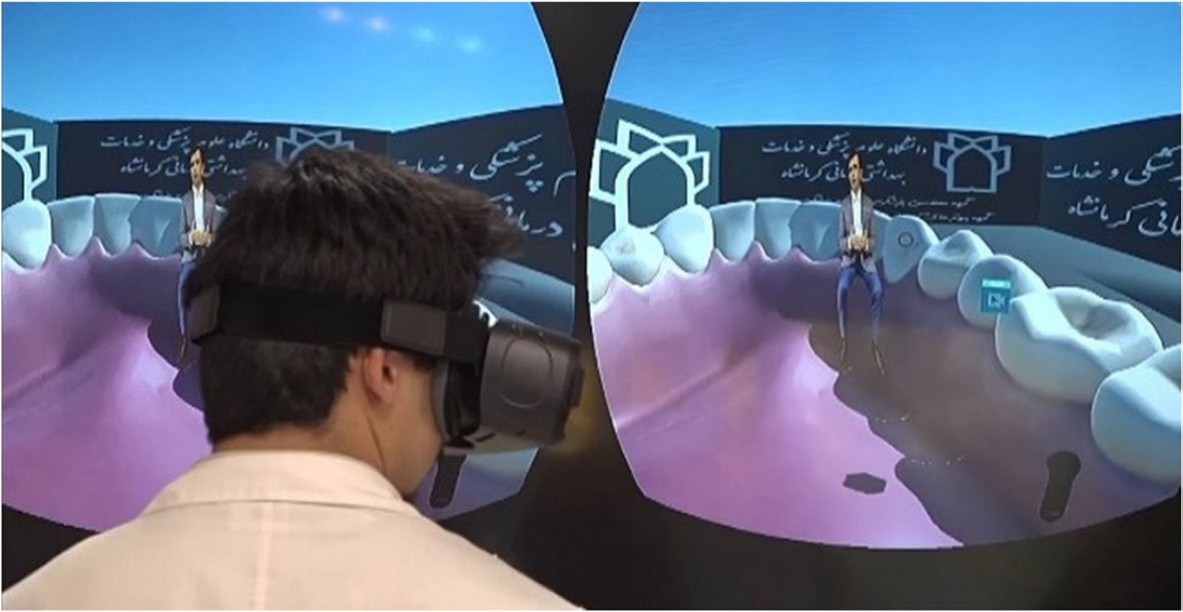
screenshot of the virtual reality environment

**Fig. 5:**
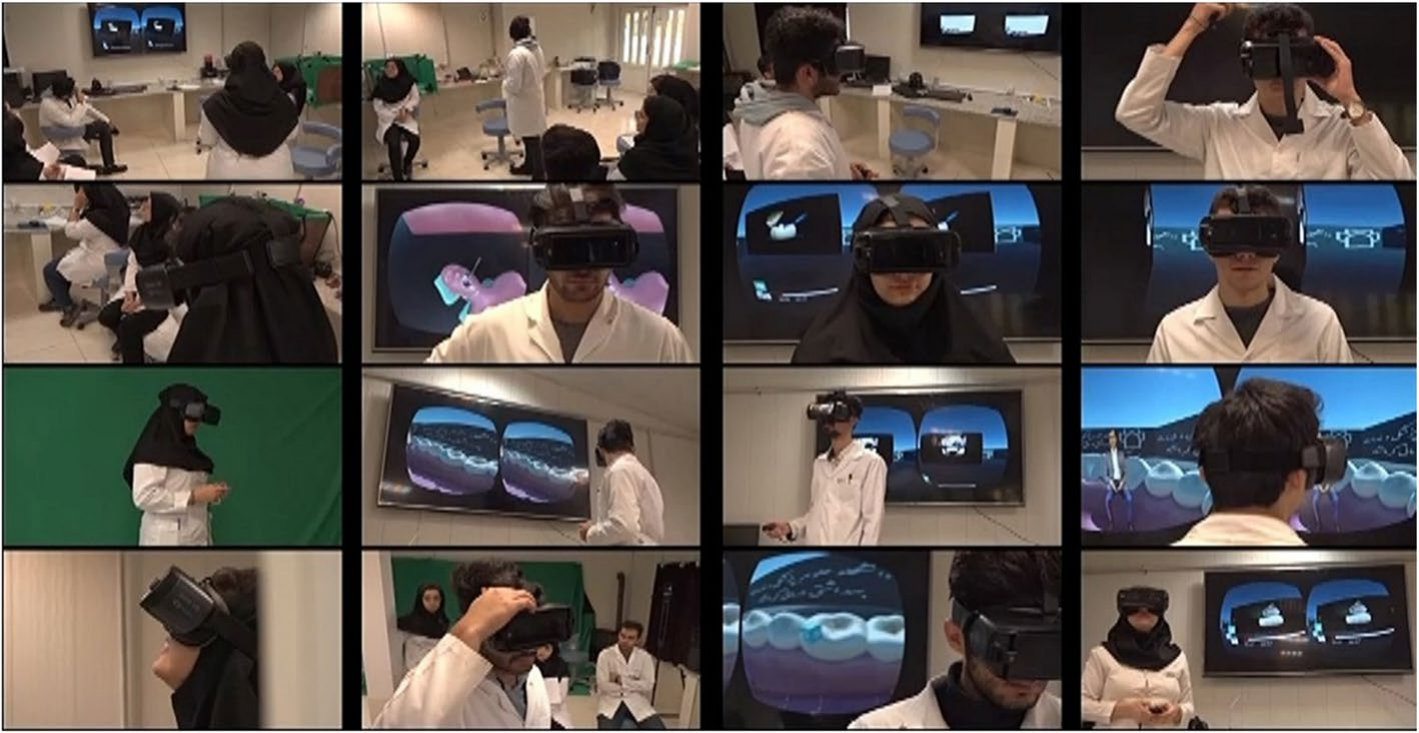
A few images of students using virtual reality headset in the learning process

After preparing the hardware and software, the green background of the camera recordings was removed to create a 3D environment using Camtasia Studio 6 and Adobe Premiere Pro software. Subsequently, a 3D cube was created using the Unity game engine, where each side of the cube just represented the video data of a single camera. Finally, by creating the mobile application for Samsung phones and the handset mounted on the student’s headset, the students were able to see and then replicate the learning procedures by rotating the cube using the headset controller (Fig. 4). A few screenshots of students using virtual reality headset in the learning process are shown in Fig. 5.

### Phase two

After designing the VR technology, a workshop entitled “application of VR in dental education” was held, and the faculty members of the department of dentistry (including 10 people) were invited to this workshop. The VR technology was introduced in this workshop, and its educational applications were explained. The VR technology was then provided to each participant to get familiar with in a completely tangible way. At the end of the workshop, a questionnaire was used to assess the strengths and weaknesses of VR technology.

**Fig. 6:**
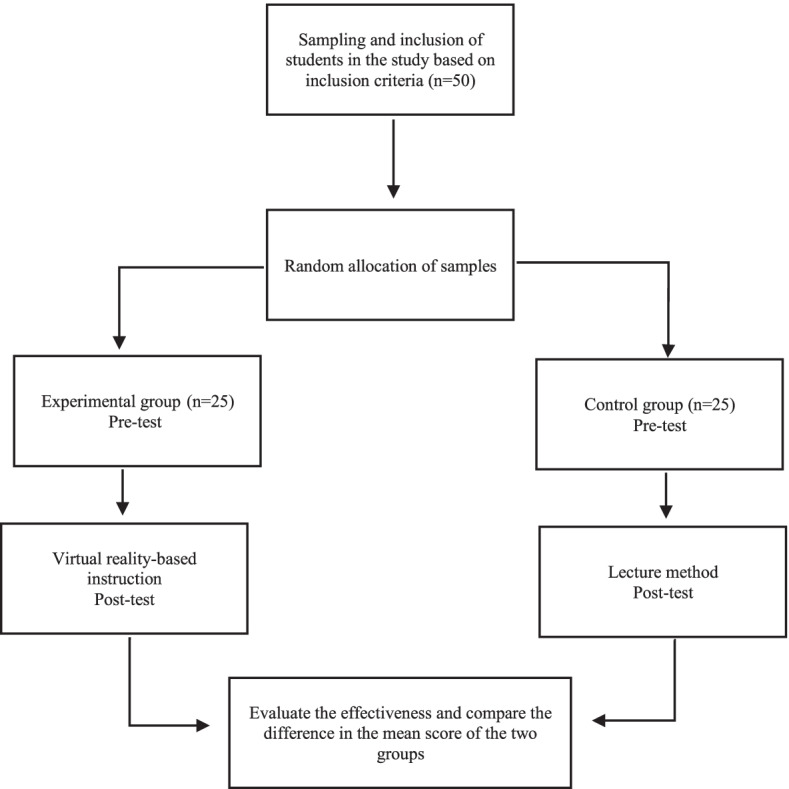
Flowchart of study

The next step was to evaluate the effect of VR technology on the academic performance of dental students. In this step, the students who were recruited in the study were divided into experimental and control groups. The VR technology was presented to the experimental group, and the traditional teaching method was presented to the control group (Fig. 6).

Both groups had the same educational objectives and were presented by the same teacher. A pre-test (before the presentation) and a post-test were used to evaluate the change in knowledge in each group. Before the course presentation, a briefing session was held on the use of the equipment and VR educational content, all the questions were answered, and the ambiguities were clarified. After training, a practical skill test on the neutral zone design and teeth arrangement was given to both groups by a blinded prosthodontist based on the course objectives.

In this study, we tried to prevent the occurrence of contamination bias. we trained the control and experimental groups in two separate classes. Therefore, students in the control group were not exposed to the intervention. An effective way to reduce pollution bias is to strengthen collaboration and dialogue with the study sample [13, 14]. In this regard, we talked to the students of the experimental group not to provide information about the virtual reality-based education method to the students of the control group until the end of the study.

### Data collection tool

A questionnaire was used to assess the faculty members’ perspectives toward the usability of VR technology in dental education. The questionnaire items were designed based on a questionnaire designed in the study of Siyah Mansoory et al. [5]. The questionnaire consisted of 11 items and was graded based on a 5-point Likert scale. The face validity and content validity of the questionnaire were confirmed by experts. Its reliability was determined by Cronbach’s alpha coefficient, which was equal to 75.

To evaluate the practical skills acquired by the students via both conventional and VR teaching techniques regarding the teeth management and base and wax rim fabrication based on the Hobrink’s reference book [15], the 6-item and 9-item checklists were used, respectively. Accordingly, these items were extracted from Hobrink’s reference book used at the dental faculty according to the dentistry curriculum [15]. These items involve the characteristics of proper teeth arrangement and base and wax rim design. This test is considered a standard test in the Iranian curriculum to assess the students’ practical works in these domains. Cohen’s Kappa coefficient was used to determine the inter-rater reliability of the test, which indicated an acceptable reliability index (0.75).

The scores ranged from 0 to 20, and the test scores of both groups were then compared. The results of the K-S test showed that the scores of both tests were normally distributed. Therefore, the t-test was used to compare the scores obtained from the clinical skills test between these the two groups. *P* = 0.05 was considered statistically significant.

To evaluate the students’ satisfaction with the course presented by the VR system, a researcher-made questionnaire, rated on the five-point Likert scale, was sued. The content validity of the questionnaire was confirmed by 10 faculty members of the school of dentistry after analyzing and revising it in several stages. The reliability of the questionnaire after completion by students was calculated to be 76% by Cronbach’s alpha, which was an acceptable level of reliability.

### Data analysis

The data collected were analyzed by the Statistical Package for Social Sciences (SPSS v.19.0; SPSS Inc., Chicago, IL, USA) using descriptive (frequency, percentage, mean and standard deviation) and inferential (Independent sample t-test) statistics. P-value of less than 0.05 was considered as significant level.

### Ethical consideration

The present study was approved by the Ethics Committee of KUMS in compliance with ethical requirements (IR.KUMS.REC.1398.719). This manuscript was registered on 2019–10-15. In this study, the principles of ethics set out in the Helsinki Declaration were taken into account. Written consent was obtained from all participants, and they were also assured that their information would be kept completely confidential. This manuscript is in accordance with CONSORT 2010 guidelines.

## Results

Out of 50 participants in this study, 52% were male (*n* = 26) and 48% (*n* = 24) were female. The mean age of students was 23.32 ± 3.36. The results of the survey from the faculty members of the dental school showed that 100% of them confirmed the usability of this technology. The mean usability of virtual reality from perspective of faculty members was 50.10 ± 4.45 out of 55 (Table 1). The results of the survey from the students showed 76% of them thought that they were completely satisfied with the VR technology and 96% of them reported a high level of comprehension of the materials presented by this technology. The total maen score of students’ satisfaction was equal to 41.12 ± 5.65 out of 45 (Table 2).

**Table 1:** Virtual reality usability of perspectives of dental faculty members

No	Item	Very good	Good	Average	Poor	Very poor
1	Possibility of multiple observations and repetitions of the topics	7(70%)	3(30%)	0	0	0
2	Possibility of focusing on the practical phantom	8(80%)	2(20%)	0	0	0
3	Possibility of administration and generalization in other educational centers	10(100%)	0	0	0	0
4	Running virtual classes by this method	5(50%)	5(50%)	0	0	0
5	Saving time	7(70%)	3(30%)	0	0	0
6	Achieving educational objectives	1(10%)	6(60%)	3(30%)	0	0
7	Learning process	1(10%)	8(80%)	1(10%)	0	0
8	Possibility of simultaneous use by several students	8(80%)	2(20%)	0	0	0
9	Possibility of commercializing this technology	7(70%)	3(30%)	0	0	0
10	Possibility of running forums for student–teacher interaction	5(50%)	4(40%)	1(10%)	0	0
11	Use of this technology in other subjects of dentistry courses	7(70%)	3(30%)	0	0	0
Total	Virtual reality usability	Mean (SD) = 50.10 ± 4.45

**Table 2:** Assessment of students’ satisfaction with the course using the VR technology

No	Component	Completely agree	Agree	Neutral	Disagree	Completely disagree
1	Using this technology is easy for me	16(24%)	8(32%)	0	1(4%)	0
2	This technology is highly reliable	10(40%)	12(48%)	2(8%)	1(4%)	0
3	Using this technology increases accuracy	19(76%)	4(16%)	1(4%)	1(4%)	0
4	Using this technology promotes comprehension	24(96%)	0	1(4%)	0	0
5	Time can be managed better by this technology	16(64%)	7(28%)	2(8%)	0	0
6	Learning is more enjoyable with this technology	21(84%)	3(12%)	1(4%)	0	0
7	I achieved the course objectives with this technology	12(48%)	11(44%)	2(8%)	0	0
8	I felt at ease by using this technology	15(60%)	7(28%)	1(4%)	2(8%)	0
9	I am completely satisfied with this technology	19(76%)	4(16%)	0	2(8%)	0
Total	Students’ satisfaction	Mean (SD) = 41.12 ± 5.65				

The results showed that the mean scores obtained in each end-of-course test were higher in the experimental group than in the control group (Table 3). The mean scores of the application of the neutral zone test showed a statistically significant difference (p = 0.021), while this difference was not statistically significant for teeth arrangement in the complete denture test (p = 0.193).

**Table 3:** Statistical analysis of pretest and posttest results of the experimental and control groups

	Subject	Group	Mean ± SD	MD^*^ ± SD	*P*-value^*^
Pretest	Neutral zone assessment	Experimental	16.14 ± 1.18	-0.50 ± 0.35	0.888
		Control	16.19 ± 1.32		
	Teeth arrangement assessment	Experimental	15.65 ± 1.17	0.10 ± 0.34	0.773
		Control	15.55 ± 1.26		
Posttest	Neutral zone assessment	Experimental	16.92 ± 1.12	0.78 ± 0.32	0.021^*^
		Control	16.14 ± 1.18		
	Teeth arrangement assessment	Experimental	16.07 ± 1.07	0.42 ± 0.31	0.193
		Control	15.65 ± 1.17		

^*^^P^.^≤0.05^

## Discussion

The role of digital technologies and technology-enhanced simulations such as VR in the education of health professions has been emphasized in many studies[9, 16]. This study evaluated the effect of VR technology on teaching neutral zone and teeth arrangement. Based on the results of the first question of the study, the majority of faculty members confirmed the usability of VR technology in the dental education process. Further, according to the results of the second question, the students showed a very high level of satisfaction with VR technology.

This part of the results of the present study is in line with the findings of some other studies. In this regard, the results of a study among Japanese dental students showed that the VR system improved students’ skills in porcelain fused to metal crown preparation. Students also expressed their satisfaction with the use of this technology in dental education [17]. In another study, Welk et al. showed that 87% of students found the VR simulators very attractive [18]. Lloréns et al. indicated the VR system was usable and motivating for users [19].

Technological advances in recent years have facilitated the use of VR technology in the dental education process. This has significantly contributed to the enrichment of the learning environment and improvement of students’ diagnostic skills. The extensive use of VR technology and simulators in dental education is owing to their significant benefits. The possibility of self-assessment, rapid acquisition of knowledge and skills by students, reducing the risks that negatively affect the patients’ health, and increasing the level of safety of students facing patients [20] are among the most important benefits of using a VR system, which has led to high satisfaction among students [21].

Realism is a very important element in e-learning. VR technology has a very high potential for creating realistic training [20]. Acquiring clinical knowledge and skills and the ability to transfer these skills to a real clinical environment in dentistry are vital necessities. Designing and presenting clinical scenarios in the VR context is a very effective opportunity to transfer clinical skills to medical students. Evidence indicates the positive effect of VR education on students’ knowledge and skills [22–24]. Providing continuous feedback to students in the preclinical environment is one of the most important strengths of VR simulators. In contrast, in traditional preclinical courses, the supervisor provides feedback at the end of the process and the end of the task [24].

The results related to the third question of the study showed that the mean score of students was significantly higher in the experimental group than in the control group in neutral zone teaching. However, in teeth arrangement teaching, although the mean score of students was higher in the experimental group than in the control group, this difference was not statistically significant.

The results of this section were in line with the findings of some other studies in terms of the effectiveness of VR technology as an educational tool. Imber et al. showed that the VR simulator was an effective way to evaluate the performance of dental students [25]. In a systematic review, Joda et al. concluded that the use of VR and augmented reality (AR) technology in dental education is increasing [26]. Moreover, VR/AR technology is also an effective tool for teaching complex procedures in dentistry. Kikuchi et al. also showed the effectiveness of the VR simulator system in improving the dental education for porcelain fused to metal (PFM) crown preparation [17]. Zafar et al. also reported that more than half of the students believed that the VR system increased their perception of pediatric dentistry [27].

Overall, the findings of our study clearly showed the effective role of VR simulators in improving the performance of dental students and increasing their satisfaction with this technology. As mentioned, the findings of other studies also confirm this[5, 28–32]. The VR simulator has a wide range of applications in the fields of patient diagnosis and treatment and teaching students and dental professionals. The VR technology as an educational tool can provide valuable opportunities in the learning-teaching process for students of health professions, including dentistry. This technology can increase the attractiveness of learning and the effective transfer of knowledge and motor skills [33, 34].

The necessity and importance of using digital technologies in medical education have become an undeniable fact. This necessity became even more pronounced during the Covid-19 pandemic [35]. The VR technology, despite its significant advantages, has challenges in medical education that need to be addressed. These challenges include reduced face-to-face communication, learning-related challenges, user attitudes, cost challenges, and teacher-student challenges [36, 37]. An accurate understanding of these challenges can play an important role in the better use of this technology in the context of dental curricula.

The present study was the first study on the application of VR technology in dental education in Iran. Based on a review of research literature, we did not find any studies in other countries on the application of VR technology in teaching neutral zone and teeth arrangement. In this study, in addition to examining the effectiveness of VR technology, we examined its usability from the perspective of faculty members and evaluated students’ satisfaction with the use of this technology in the learning process.

The use of any new technology in the learning process has challenges for faculty members. Therefore, technical and educational support for instructors is very important. Virtual reality technology is a complement. It is also not a substitute for expert instructors. Therefore, it should be used along with other main teaching methods. This will compensate for the lack of traditional teaching methods. Combining virtual reality technology with other new technologies such as 5G Internet and big data has an effective role in creating deeper learning for students. Due to the high capability of virtual reality technology, this technology can be used in teaching many skills such as dental surgery, periodontics, endodontics, implantology [38].

We used a gear headset in this study. Future studies are suggested to use headsets or glasses that allow the user to interact more with educational elements and movement in a simulated environment.

This study faced some limitations. One of the limitations was the lack of familiarity of students and faculty members with the VR technology. To this end, the researchers held a workshop on the introduction and application of VR technology for the participants. The reason for the unfamiliarity of the participants was that the VR technology in Iranian medical universities is completely new. Therefore, very few universities have access to this technology. Another limitation of the present study was the students’ feeling of confusion after using a VR headset. Moreover, students who wore glasses could not use a VR headset, so they were excluded from the study and were replaced with other participants.

## Conclusion

This study investigated the effectiveness of VR technology in teaching neutral zone and teeth arrangement in Iran. The results indicated the acceptable usability of this technology in dental education. In addition, dental students had good satisfaction with the application of VR technology in the teaching process. The most important finding of this study was that the mean score of students was significantly higher in the VR technology teaching group than in the traditional teaching group.

Teaching health professions such as medicine and dentistry is changing. Therefore, the use of new technologies in these areas has become a vital necessity. However, VR technology should not be thought of as a panacea [39]. The VR technology is only a complementary educational tool and can play an important role in improving the quality of teaching and improving students’ skills if it is defined in the framework of the curriculum and its pedagogical dimensions are known. Finally, we believe that although the use of VR simulators in medical and dental education is increasing in developed and developing countries, further research is needed to gain a more accurate understanding of its effectiveness, challenges, and opportunities.

## Data Availability

Data and materials are available by contacting the corresponding author.

## Abbreviations

VR: Virtual reality
AR: Augment reality
PFM: Porcelain fused to metal
3D: 3-Dimensional
